# Resveratrol ameliorates the behavioural and molecular changes in rats exposed to uninephrectomy: role of hippocampal SIRT1, BDNF and AChE

**DOI:** 10.1007/s13105-022-00937-x

**Published:** 2022-12-05

**Authors:** Marianne Basta, Samar. R. Saleh, Rania. G. Aly, Abeer. E. Dief

**Affiliations:** 1 Department of Medical Physiology, Faculty of Medicine, University of Alexandria, Fahmy Abd Elmagid St., Alexandria 21131 Elhadara, Egypt; 2 Department of Biochemistry, Faculty of Science, Alexandria University, Baghdad St., Moharam Bek, Alexandria, 21511 Egypt; 3 Department of Pathology, Faculty of Medicine, University of Alexandria, Fahmy Abd Elmagid St., Alexandria 21131 Elhadara, Egypt

**Keywords:** Uninephrectomy, Resveratrol, SIRT1, Behavioural, Memory

## Abstract

Subtle memory and cognitive changes may occur in uninephrectomized (Unix) patients long before the development of chronic kidney disease, such changes may be unnoticed. The dietary polyphenol, Resveratrol, displayed various neuroprotective effects, its role in chronic kidney disease is an area of intense studies. This work was designed to investigate the behavioural and molecular changes that may occur following 7 months of Unix in rats, and to determine whether Resveratrol intake can improve such pathology. Male Wistar rats were divided into three groups: sham operated, Unix and Unix group treated with Resveratrol (20 mg/kg/day). Rats were subjected to series of behavioural testing, different biochemical parameters along with RT-PCR and immunohistochemistry of the hippocampal tissue to track the development of functional or structural brain changes. Anxiety behaviour and reduced spatial memory performance were observed in rats 7 months post-nephrectomy; these deficits were remarkably reversed with Resveratrol. Among the species typical behaviour, burrowing was assessed; it showed significant impairment post-nephrectomy. Resveratrol intake was almost able to increase the burrowing behaviour. Decreased SIRT1 in immune-stained sections, oxidative stress, inflammatory changes, and increased AChE activity in hippocampal homogenates were found in Unix rats, and Resveratrol once more was capable to reverse such pathological changes. This work has investigated the occurrence of behavioural and structural brain changes 7 months following Unix and underlined the importance of Resveratrol to counterbalance the behavioural impairment, biochemical and brain pathological changes after uninephrectomy. These findings may raise the possible protective effects of Resveratrol intake in decreased kidney function.

## Introduction

Cognitive impairment is common in patients with renal insufficiency revealing a kidney–brain cross-talk. Decline in cognitive functions even appears early in mild and moderate kidney diseases before progression to end-stage renal disease (ESRD). Cognitive impairment in renal patients not only includes learning and memory impairment but also extends to executive dysfunction, attention-deficit and verbal difficulty [10].

In early stages of renal dysfunction, albuminuria represents systemic vascular burden causing both cognitive and brain structural changes [10]. Risk factors also include the increased systemic oxidative stress [29], and inflammation that alters the blood–brain barrier structure, increasing its permeability to noxious substances, e.g. uremic toxins and inflammatory cytokines [10]. Additionally, dyslipidaemia, hypertension, microvascular and cardiovascular events occurring in renal diseases represent another burden [29].

Changes in brain structure and neurotrophic factors account for impaired cognitive functions in renal dysfunction, e.g. decrease in brain-derived neurotrophic factor (BDNF) expression [29]. A recent study showed that kidney dysfunction leads to decreased acetyl cholinesterase (AChE) activity in different brain regions with increased oxidative stress, inflammation and mitochondrial activities in some parts of the brain. Altogether, accompanied with neuronal changes such as decreased arborization and dendritic spine density and activated glial cells [19].

Decreased renal reserve in uninephrectomized (Unix) animals was followed by manifested proteinuria, increased serum urea and creatinine and significant fibrosis of the remaining kidney at 7 months post-nephrectomy [2]. Similarly, humans who underwent nephrectomy started to show insufficient renal compensation within 6 months of the surgery, manifested by albuminuria and increased serum creatinine. The rate of albuminuria increased with time and was associated with renal disease progression that could end finally in ESRD [24]. Moreover, the renal changes following nephron loss were accompanied with metabolic [2] and cardiovascular changes, and the manifested albuminuria could lead to vascular insults [24]. Generally, rats showed rapid renal and systemic changes in response to Unix, and what happened in few months in the remaining kidney of rats could take 20 years or more in humans [2]. Therefore, we assumed that Unix rats might show cognitive and brain molecular changes at 7 months post-nephrectomy.

The widespread polyphenol, Resveratrol, has ameliorated the renal structural and functional changes occurring 7 months post-nephrectomy in adult male Wistar rats either through its anti-oxidative properties or SIRT1 activation [2]. Besides, Resveratrol showed neuroprotective properties in neurological diseases, e.g. Alzheimer’s, decreasing the neuronal changes, the inflammatory, oxidative and vascular insults [9]. Therefore, we assume that Resveratrol administration to Unix rats may ameliorate the cognitive and brain molecular changes that may follow the procedure either through its reno-protective or direct neuroprotective effects. However, there has been some disagreement concerning the neuroprotective effect of Resveratrol [27]. Therefore, this study focuses on the behavioural changes that may develop in rats after long-standing nephrectomy, and to correlate the encountered changes to molecular mechanism, then discover if Resveratrol treatment would be of value to ameliorate these changes.

## Methods

### Animals, husbandry and surgical procedures

The study protocol was approved by the Research Ethics Committee of Alexandria Faculty of Medicine, approval number 0305128. Animals were provided from Animal House of Medical Physiology, Faculty of Medicine, University of Alexandria Twenty-six adult male Wistar rats (12–14 weeks old, 100–150 g body weight) were included. After 1 week of acclimatization, rats were weighed and randomly assigned into 3 subgroups: control (Sham-operated, *n* = 6), uninephrectomized (Unix, *n* = 10), and uninephrectomized treated with Resveratrol (Unix + Res, *n* = 10). All rats had free access to food (standard laboratory chow; 3% fat, 15% protein, 50% polysaccharide, 7% simple sugars and vitamins and minerals; energy 3.5 kcal/kg) and water and were kept under controlled 12-h light–dark cycles.

Rats in Unix and Unix + Res groups underwent surgical removal of the right kidney as described [2], whereas control rats were subjected to the same procedure without kidney excision. After 1-week recovery, all rats were weighed and rats in Unix + Res group received 20 mg/kg of Resveratrol by oral gavage every day till the end of the study (7 months post-nephrectomy) [2]. Oral gavage was conducted by an efficient lab personnel daily at the same time of the day (09:00–11:00 am). Resveratrol was obtained from Carbosynth, San Diego, U.S.A (98% purity, formula: C_14_H_12_O_3_).

Rats in the other two groups received an equivalent amount of vehicle, orally by gavage, on daily basis till the end of the experiment [2]. During the experiment, one rat died from sham-operated group and two rats also from each of the other two groups.

### Behavioural tests

All behavioural tests were performed between 9 am and 1 pm by a researcher blinded to the groups’ identity.

#### Short-term spatial working memory

##### T maze alteration

The T maze was placed properly, and the guillotine doors were raised. Rats were placed in the start area and allowed to choose between the two goal arms. After going into the chosen arm, the door was set down and the animal was kept in place for 30 s. Then, the rat was removed, placed in the start area again with the 3 doors open and allowed to choose between the two goal arms again. Each trial was kept at a maximum of 2 min. The test was repeated for ten trials, and the percentage of correct alterations was counted [12].

#### Species typical behaviour

##### Burrowing

The rats were placed with a burrow which is cylindrical plastic tube around 20 cm long and 6 cm width, plugged at one end, individually in the cage. The burrow was filled with 200 g of rat chow, and the rats allowed to burrow for 2 h; after that, the remaining chow in the burrow was weighed to estimate the burrowed chow by the rat. Estimation of the weight burrowed over 24 h was performed on a separate day [12].

#### Emotionality and exploratory behaviour

##### Light–dark box (LDB)

Rats were placed in lit compartment and allowed to freely move between both compartments. The duration spent in lit part, number of transitions between the two compartments, and latency before returning to lit part were all recorded and their percentages were calculated [20].

##### Open field test

The open field was a 50 × 30 × 18 cm black arena, alienated into 10 × 10 cm squares. This test is used as a measure for anxiety, besides being extremely sensitive to both activity and exploration. Each rat was placed in a corner square, confronting the walls, and observed for 3 min. The following parameters were recorded: latency to leave the first square, latency to the first rear, the total number of squares crossed and the total number of rears [12].

### Urine collection and study termination

Twenty-four-hour urine was collected 1 week before termination. Each rat was placed individually in a metabolic cage for 24 h, with free access to chow and water. The collected urine was centrifuged at 3000 × *g* for 10 min before being stored at − 80 °C [2].

The study was terminated at 7 months post-surgery. On the day of termination, rats were fasted for 12 h, weighed (terminal body weight, TBW) and killed via intraperitoneal injection of lethal dose of anaesthesia (200 mg/kg of ketamine and 10 mg/kg of xylazine). Blood was collected by cardiac puncture and left kidneys were collected and weighed. The left kidney weight/TBW% was calculated [2]. The whole brain was collected and washed with ice-cold saline. One hemisphere was fixed with formalin for histological examination. The hippocampal tissues were quickly dissected from the other hemisphere and stored at − 80 °C for protein extraction and biochemical analysis [14].

### Biochemical studies

Serum and urine creatinine were measured by the Jaffe kinetic method, whereas serum urea was measured by the Berthelot enzymatic method (Diamond Diagnostics, Schiff graben, Hannover, Germany). Urine protein concentration was measured by the pyragallol red/SDS method (Wellkang Ltd, Harley Street, London, UK) [2].

Fasting serum glucose, triglycerides, total and HDL cholesterol (HDL-C) were measured in terminal samples using enzymatic methods (BioSystems S.A., Costa Brava, 30. 08,030 Barcelona, Spain). Kits were used according to the manufacturers’ instructions. Non-HDL cholesterol (non-HDLc) was calculated using the following formula, total cholesterol–HDL-C. Atherogenic index was calculated as (total cholesterol − HDL-C)/HDL-C [2].

### RNA isolation and reverse transcriptase polymerase chain reaction (RT-PCR) in hippocampal tissues

Total RNA was isolated from hippocampal tissues (approximately 100 mg) using GENEzol™ Reagent (Geneaid, Taiwan). The concentration and purity of RNA was estimated by using a NanoDrop 2000 spectrophotometer (Thermo Scientific, USA). The absorbance was measured at 260 and 280 nm. Samples with A260/280 ≥ 1.8 were used further. Isolated RNA was reverse transcribed into complementary DNA (cDNA) using Viva cDNA synthesis kit (Vivantis, Malaysia). PCR reaction was amplified using cDNA as template and glyceraldehyde-3-phosphate dehydrogenase (GAPDH) as housekeeping gene. Primers sequence for GAPDH [18], BDNF [11], cyclic AMP response element binding protein (CREB) [11], AChE [11], interleukin 1 beta (IL-1β) [23], tumour necrosis factor alpha (TNF-α) [35], indoleamine-2,3-dioxygenase (IDO) [18], inducible nitric oxide synthase (iNOS) [18], insulin growth factor 1 (IGF 1) [1], transforming growth factor beta (TGF-β) [6] and peroxisome proliferator activated receptor gamma (PPARγ) [34] are shown in Table 1. PCR mixture was prepared as follows: 10 µl of Taq qPCR Green Master Mix (Vivantis, Malaysia), 1 µl of forward primer, 1 µl of reverse primer and 1 µl of template cDNA was dispensed in PCR tubes (0.2 ml) then completed to 20 µl with nuclease-free distilled water. PCR was performed using the following thermal cycling conditions: initial denaturation at 95 °C for 2 min, 40 cycles of denaturation at 95 °C for 15 s, annealing as shown in Table 1 and extension at 60 °C for 30 s. qRT-PCR was performed using CFX96™ Real-Time System (Bio-Rad, USA). The quantities critical threshold (Ct) of target gene was normalized with quantities (Ct) of housekeeping gene (GAPDH) by using the 2^−∆∆Ct^ method to calculate the fold change in target gene.

**Table 1:** RNA isolation and RT-PCR (primers’ sequences and annealing temperature)

Name	Forward	Reverse	Annealing Tm (°C)
GAPDH	AGATCCACAACGGATACATT	TCCCTCAAGATTGTCAGCAA	52
PPAR γ	TGATATCGACCAGCTGAACC	GTCCTCCAGCTGTTCGCCA	60
BDNF	GGACATATCCATGACCAGAAAGAAA	GCAACAAACCACAACATTATCGAG	60
CREB	CTGATTCCCAAAAACGAAGG	CTGCCCACTGCTAGTTTGGT	60
AChE	TTCTCCCACACCTGTCCTCATC	TTCATAGATACCAACACGGTTCCC	52
IL-1β	TGCCACCTTTTGACAGTGAT	TGTGCTGCTGCGAGATTTGA	52
TNF-α	CCCCAAAGGGATGAGAAGTTC	GGCTTGTCACTCGAATTTTGAGA	52
IDO	AGAAGTGGGCTTTGCTCTGC	TGGCAAGACCTTACGGACATCTC	60
iNOS	AAGGACTATCTCCACCAGG	CCTCATGATAACGTTTCTGGC	60
IGF 1	CAG ACA GGA GCC CAG GAA AG	AAG TGC CGT ATC CCA GAG GA	52
TGF-β	TGA GTG GCT GTC TTT TGA CG	TCTCTGTGGAGCTGAAGCAA	52

Primer sequences for glyceraldehyde-3-phosphate dehydrogenase (GAPDH), brain-derived neurotrophic factors (BDNFs), cyclic AMP response element binding protein (CREB), acetyl choline esterase (AChE), interleukin 1 beta (IL-1β), tumour necrosis factor alpha (TNF-α), indoleamine-2,3-dioxygenase (IDO), inducible nitric oxide synthase (iNOS), insulin growth factor 1 (IGF 1), transforming growth factor beta (TGF-β) and peroxisome proliferator activated receptor gamma (PPARγ) are shown

### Assessment of acetylcholine esterase (AChE) activity assay in hippocampal homogenates

The activity of acetylcholine esterase was determined according to the method of Ellman, Courtney [7]. Acetylcholine thioiodide (ACTI) was used as artificial substrate. The reaction mixture (190 µl final volume) contained 130 μl of phosphate buffer (pH 8.0), 20 μl of 5,5-dithiobisnitrobenzoic acid (DTNB, 0.32 mM) and 20 µl of ACHI (20 mM). The assay was started by adding 20 μl of brain homogenate supernatant, and the increase in the absorbance was recorded at 412 nm for 3 min at room temperature with 60-s intervals. The control was performed using 20 μl of phosphate buffer (pH 8.0) instead of the supernatant. The specific activity is expressed in micromoles of ACHI hydrolysed/min/mg protein (µmol/min/mg protein). Total protein (mg/ml) was estimated by the method of Lowry, Rosebrough [17] for determination of AChE specific activity.

### Assessment of malondialdehyde (MDA) level in hippocampal homogenates

MDA, which is a major secondary product of lipid peroxidation, was measured by using the thiobarbituric acid assay as describe by Tappel and Zalkin [31]. Briefly, 500 µl of hippocampal homogenate supernatant (test) or water (blank) were mixed with 1 ml trichloroacetic acid (15%) and centrifuged at 3000 rpm for 10 min. About 0.5 ml of thiobarbituric acid (0.7%) was added to 1 ml of the supernatant, boiled for 60 min to allow chromophore development. The absorbance was read at 532 nm against blank. The MDA content was calculated using a molar extinction coefficient of 1.56 × 10^−5^ M^−1^ cm^−1^. The results were expressed as µmol/mg protein, respectively.

### Brain histology and immunostaining

The brain tissues from the different groups were fixed in 10% formaldehyde and embedded in paraffin blocks for histopathological investigations. They were stained with hematoxylin and eosin (H&E) and examined using the light microscope (Leica, Germany). IHC using SIRT-1 (monoclonal antibody, B-10: Sc 79,504) (Santa Cruz biotechnology, USA) of all sections was done using the avidin–biotin-peroxidase method [13]. The antibody was added to each section using the Bond-Max fully automated immunostainer (Leica Biosystems, USA). The quantification of the IHC was done, in each slide, using the quantitative-image analysis (Leica micro-systems, Switzerland). Human testis was used as positive control for SIRT-1 antibody, according to the manufacturer datasheet, and included in all runs. Negative control sections, by omitting the primary antibody, were also included in all runs.

### Statistical analysis

Data are presented as means ± SEM. Shapiro–Wilk test was used to test for normality. One-way ANOVA was used for evaluation of results, followed by post hoc pairwise analysis without adjustment for multiple comparisons. For skewed variables, Log-10 transformations were made. All tests were two-tailed, with *P* < 0.050 being considered significant. SPSS Statistics for Windows (20.0; SPSS Inc., Chicago, IL, USA) was conducted for analysis.

## Results

### Effect of Resveratrol on kidney weights and functions in Unix rats

At 7 months post-surgery, Unix induced a decrease in both TBW (9% decrease, *P* = 0.131) and weight gain (15% decrease, *P* = 0.156), compared to control. Similarly, Resveratrol administration in Unix rats significantly decreased TBW (*P* = 0.004) and weight gain (*P* = 0.008), compared to control. On the other hand, Unix rats showed a significant increase in left kidney weight/TBW %, compared to sham operated rats (68% increase, *P* < 0.0001). Controversially, Unix rats treated with Resveratrol showed significant lower percentage than untreated Unix rats (≤ 20% lower, *P* < 0.0001), but significant higher ratio than control ones (38% higher, *P* < 0.0001), Table 2.

**Table 2:** Effect of Resveratrol on kidney weights and functions, and body weight and metabolism in Unix rats

Variable	Control	Unix	Unix + Res
Terminal body weight (TBW) (g)	290 ± 13.5	264 ± 7	235 ± 13*
Weight gain (g)	156 ± 13.3	132 ± 7.6	108 ± 11.7*
Left kidney weight/TBW %	0.31 ± 0.0005	0.52 ± 0.02**	0.43 ± 0.001**^, ##^
Serum urea (mg/ dl)	42.1 ± 2.4	58.6 ± 2.90*	48.4 ± 2.6 ^#^
Serum creatinine (mg/ dl)	0.98 ± 0.04	1.26 ± 0.04**	1.08 ± 0.02 ^##^
24-h urine total protein(mg)	2.8 ± 1.2	7.9 ± 1.2**	4.6 ± 1.1*^, #^
Urine protein/creatinine ratio	0.65 ± 0.14	1.2 ± 0.23*	0.78 ± 0.15
Fasting serum glucose (mg/dl)	77.2 ± 2.8	97.5 ± 1.8*	83.5 ± 5 ^#^
Fasting serum triglycerides (mg/dl)	70.2 ± 2.4	87.2 ± 3.6*	74.2 ± 2.5 ^#^
Fasting serum cholesterol (mg/dl)	87.1 ± 6.8	109.2 ± 6*	84.9 ± 4.6 ^#^
Fasting serum HDL (mg/dl)	61.9 ± 5.1	61.8 ± 3.2	59.6 ± 2.8
Fasting serum non HDLc (mg/dl)	24.93 ± 1.1	45.78 ± 1.1*	22.8 ± 1.2 ^#^
Atherogenic index	0.41 ± 0.1	0.74 ± 0.15*	0.38 ± 0.03 ^#^

Data are expressed as mean ± SEM. SEM = standard error of mean, control (sham operated, *n* = 5); Unix = uninephrectomized (*n* = 8); Unix + Res = uninephrectomized-treated with Resveratrol (*n* = 8). *P* values are from one-way ANOVA

^*^P < 0.050 and **P < 0.001 respectively versus control

^#^*P* < 0.05 and ^##^*P* < 0.001, significant difference between Unix and Unix + Res rats, respectively

Unix exerted significant increase in serum urea (39% increase, *P* < 0.0001) and creatinine levels (29% increase, *P* = 0.001), compared to sham operation. Resveratrol administration was able to normalize serum urea (17.4% lower, *P* = 0.0124) and creatinine (15.4% lower, *P* = 0.0002) levels, compared to untreated Unix rats. Similarly, Unix rats showed significant higher values for 24-h urine protein (128% increase, *P* = 0.0003) and urine protein /creatinine ratio (85% increase, *P* = 0.016), compared to sham-operated rats. Resveratrol-treated rats showed significant lower urine protein values (42% lower, *P* = 0.0197) than Unix-untreated rats, but values were still significantly higher than the control ones (64% higher, *P* = 0.03). Also, Resveratrol tended to decrease the protein/creatinine ratio by about 30% than in untreated Unix rats but with a nonsignificant trend (*P* = 0.0628), Table 2.

### Effect of Resveratrol on body metabolism in Unix rats

Fasting serum glucose (26% higher, *P* = 0.035) triglycerides (24% higher, *P* = 0.0018), total cholesterol (25% higher, *P* = 0.0195) and non-HDLc (84% higher, *P* = 0.0131) levels were significantly elevated in Unix rats compared to control rats. Resveratrol treatment managed to decrease serum glucose (14% lower than Unix rats, *P* = 0.0238), triglycerides (15% lower than Unix rats, *P* = 0.0052), total cholesterol (22% lower than Unix rats, *P* = 0.0048) non-HDLc (51% lower than Unix rats, *P* = 0.0021) levels in Unix-treated rats back to normal values.

The atherogenic index was calculated and results showed significant increase in Unix rats, compared to sham operated ones (80% higher, *P* = 0.0179). Resveratrol treated rats showed a near-normal atherogenic index (36.3% lower than UNX rats, *P* = 0.0042). The level of HDL cholesterol showed non-significant changes among the studied groups (*P* = 0.994 for Unix versus control and *P* = 0.629 for Unix versus Unix + Res), Table 2.

### Unix induced deficits in memory and species-typical behaviours that was restored by Resveratrol

**Fig. 1:**
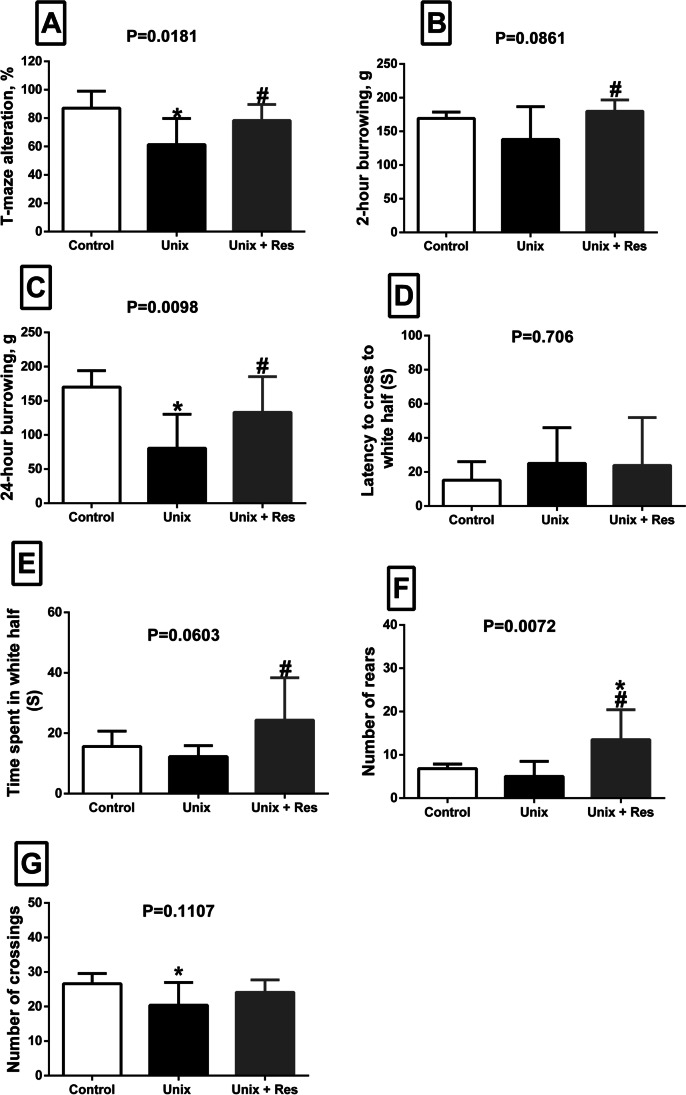
Behavioural tests. T maze alteration, % (**A**), 2-h burrowing, g (**B**), 24-h burrowing, g (**C**), LDB, latency to cross to white half (S) (**D**), LDB, time spent in white half (S) (**E**), Open field, number of rears (**F**), Open field, number of crossings (**G**) in control rats (*n* = 5), uninephrectomized rats (Unix, *n* = 8), and uni-nephrectomized rats with Resveratrol treatment (Unix + Res, *n* = 8), at 7 months post-surgery. Data are means ± SEM. P values are from one-way ANOVA. For skewed variables, Log-10 transformations were made. * *P* < 0.05 relative to control. # Significant difference between Unix and Unix + Res rats at *P* < 0.05

Unix was associated with impairment in the spatial memory as demonstrated by decreased spontaneous alternation in T maze (60% correct percentage alternation compared to control, *P* = 0.0077). Resveratrol-treated rats exhibited significantly high percentage alternation 80% which was close to the control values (*P* = 0.0491 versus untreated Unix rats), Fig. 1A. There was a tendency to decreased burrowing activity (2 h) after Unix that was normalized after Resveratrol intake (P = 0.0358 versus untreated Unix rats). Twenty-four-hour burrowing activity showed significant decrease after Unix (*P* = 0.0033) that almost normalized toward control values by Resveratrol (*P* = 0.0482 versus untreated Unix rats), Fig. 1B, C.

### The effect of Unix on anxiety and exploratory behaviour

Increased anxiety behaviour after Unix was illustrated by tendency to increased latency to cross to the white half in the LDB (25 s in Unix group versus 15 s in control group, *P* = 0.427). Time spent in the white half tended to be prolonged after Resveratrol intake by 8.7 s compared to control rats (*P* = 0.099) and 12 s compared to Unix rats (*P* = 0.02), Fig. 1D, E.

Decreased rat exploratory behaviour after Unix was manifested by tendency to less rearing behaviour (5 versus 7 in control group, *P* = 0.481) and decreased number of square crossing (20 versus 27 in control group, *P* = 0.0447) in the open field area. Rats treated with Resveratrol showed significant better results regarding number of rears compared to Unix rats (*P* = 0.022) and sham-operated rats (*P* = 0.002), and a tendency to increased number of crossings towards normal values (24 versus 20 in Unix group, *P* = 0.180), Fig. 1F, G.

### Effect of Resveratrol on SIRT 1 protein expression in hippocampal immune stained histological sections of Unix rats

**Fig. 2:**
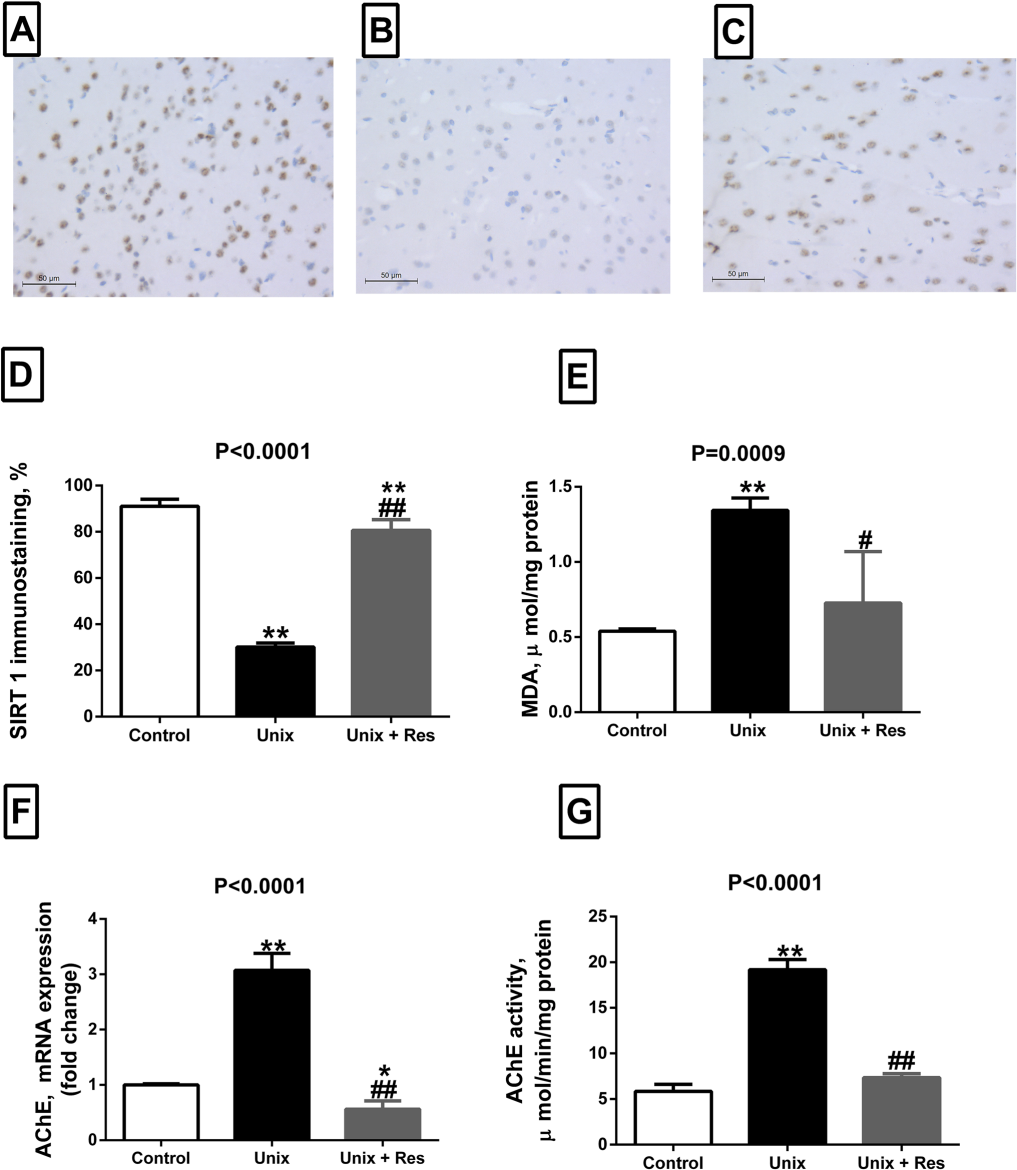
A-D, SIRT1 changes, localization, and extent of SIRT1 immunostaining in hippocampal tissue at 7 months post-surgery in control rats, uninephrectomized rats (Unix), and uninephrectomized rats with Resveratrol treatment (Unix + Res) (**A**-**C**). Image analysis of the findings shown in **A**-**C** (original magnification × 400) (**D**). MDA concentration, µmol/ mg protein (**E**), AChE mRNA expression, fold change (**F**), AChE activity assay, µmol/ min / mg protein (**G**), in hippocampal homogenate of control rats, uninephrectomized rats (Unix), and uninephrectomized rats with Resveratrol treatment (Unix + Res). Data are means ± SEM from *n* = 5 per group. *P* values are from one-way ANOVA. *, ** *P* < 0.05 and *P* < 0.001 respectively relative to control. #, ##, significant difference between Unix and Unix + Res rats at *P* < 0.05 and *P* < 0.001 respectively

Immunostaining of brain histological sections with SIRT1 antibody showed that the control group had a high distribution of SIRT-1 nuclear staining in the hippocampus, Fig. 2A–D, compared to the Unix group which showed few scattered positive cells (60% decrease, *P* < 0.0001), Fig. 2B–D. Rats treated with Resveratrol showed increased positivity compared to Unix rats (55.5% increase, *P* < 0.0001), but decreased positivity than controls (10.4% decrease, *P* < 0.0001), Fig. 2C, D (IHC, × 400).

### Effect of Resveratrol on MDA expression, and both AChE mRNA expression and activity assay in Unix rats

The lipid peroxidation marker (MDA) was significantly elevated in hippocampal homogenates of Unix rats, compared to control rats (160% increase, *P* = 0.0006). Resveratrol treatment lowered MDA levels in hippocampal homogenates of Unix rats back to control values (46% decrease versus untreated Unix rats, *P* = 0.001), Fig. 2E.

AChE mRNA expression in hippocampus was significantly upregulated in Unix rats by about threefold, compared to sham-operated rats (*P* < 0.0001). Treatment of Unix rats with Resveratrol significantly downregulated the mRNA expression of AChE, compared to both control (0.5-fold, *P* = 0.03) and Unix untreated rats (2.5-fold, *P* < 0.0001), Fig. 2F.

Similarly, AChE specific activity in hippocampal homogenates was significantly elevated in in Unix rats, compared to control ones (≥ 220% increase, *P* < 0.0001). Resveratrol treatment managed to decrease the specific activity back to normal values (62% decrease versus untreated Unix rats, *P* < 0.0001), Fig. 2G.

### Effect of Resveratrol on TNF-α, IL-1β, IDO, and iNOS mRNA expression in Unix rats

**Fig. 3:**
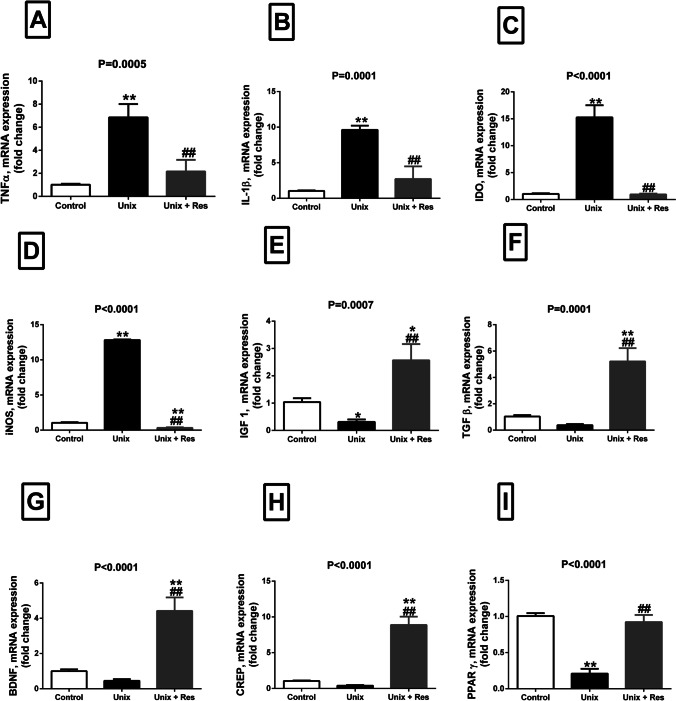
RT-PCR in hippocampal tissues. Fold change of mRNA expression of TNF-α (**A**), IL-1β (**B**), IDO (**C**), iNOS (**D**), IGF 1 (**E**), TGF-β (**F**), BDNF (**G**), CREP (**H**), and PPAR γ, in hippocampal homogenate of control rats, uninephrectomized rats (Unix), and uninephrectomized rats with Resveratrol treatment (Unix + Res). Data are means ± SEM from *n* = 5 per group. *P* values are from one-way ANOVA. *, ** *P* < 0.05 and *P* < 0.001 respectively relative to control. #, ##, significant difference between Unix and Unix + Res rats at *P* < 0.05 and *P* < 0.001 respectively

Unix rats showed significant upregulation of mRNA expression of inflammatory markers: TNF-α (> fivefold, *P* = 0.0002), IL-1β (> eightfold, *P* < 0.0001), IDO (14 folds, *P* < 0.0001) and iNOS (> 11 folds, *P* < 0.0001) in hippocampal tissues, compared to sham-operated rats. Compared to Unix-untreated rats, Resveratrol administration significantly downregulated the mRNA expression of TNF-α (4.5-fold, *P* = 0.0007), IL-1β (sevenfold, *P* = 0.0002) and IDO (14-fold, *P* < 0.0001) in hippocampal tissues, back to normal values, Fig. 3A, B and C. The mRNA expression of iNOS in hippocampal tissues was also downregulated in Resveratrol treated rats, compared to both untreated Unix (12.5-fold, *P* < 0.0001) and sham-operated rats (0.7-fold, *P* < 0.0001), Fig. 3D.

### Effect of Resveratrol on IGF 1 and TGF-β mRNA expression in Unix rats

The mRNA expression of IGF 1(by about 0.7-fold, *P* = 0.0458) and TGF-β (by about onefold, *P* = 0.216) was downregulated in Unix rats, compared to control ones. Resveratrol administration significantly upregulated IGF 1 (*P* ≤ 0.0019) and TGF-β (*P* ≤ 0.0001) mRNA expression, compared to both untreated Unix and sham-operated rats, Fig. 3E, F.

### Effect of Resveratrol on BDNF, CREB and PPARγ mRNA expression in Unix rats

Though both BDNF and CREB mRNA expression was downregulated in Unix rats compared to sham-operated ones, this did not show a significant trend (≥ 0.5-fold, *P* > 0.05). Unix rats treated with Resveratrol showed significant higher values of fold change for mRNA expression of BDNF and CREB, compared to both sham-operated and Unix-untreated rats (≥ fourfold, *P* < 0.0001), Fig. 3G, H.

Unix rats showed significant downregulation of PPARγ mRNA expression in hippocampal homogenates, compared to sham operated rats (≥ 0.5-fold, *P* < 0.0001). Resveratrol administration was able to upregulate PPARγ mRNA expression in Unix-treated rats back to control values (*P* < 0.0001 versus untreated Unix rats), Fig. 3I.

## Discussion

The risk of chronic kidney disease development in healthy kidney donors has been aroused since the last decade. Multiple studies stated that kidney donors started to show renal changes of the remaining kidney 6 months post-nephrectomy. Donors manifested decreased creatinine clearance and glomerular filtration rate, with marked albuminuria and elevated serum creatinine. Further, human kidney donors showed an eightfold higher risk of ESRD [24]. The present study showed that Unix rats developed renal and metabolic changes at 7 months post-nephrectomy, with remaining kidney hypertrophy, significant proteinuria, increased serum urea and creatinine, hyperglycaemia, hyperlipidaemia, increased atherogenic index and decreased body weight [2].

Rats in the present study showed that 7 months post-nephrectomy was accompanied by multiple behavioural changes, e.g. memory deficits, anxiety, decreased explorative behaviour and atypical species behaviour. The behavioural changes in Unix rats were escorted with increased oxidative (MDA) and inflammatory changes, as well as AChE mRNA expression and activity in hippocampal homogenates of Unix rats, together with decreased SIRT1 immunostaining.

Moreover, we also reported that Resveratrol administration to Unix rats not only ameliorated the renal and metabolic changes, but also resulted in near-normal outcomes in behavioural studies. The nephroprotective effect of Resveratrol is well known in both acute and chronic kidney diseases, e.g. diabetic nephropathy and nephrectomy. Studies related the renal protective effects to its antioxidative and anti-inflammatory effects, SIRT1 activation and metabolic regulation [2].

Additionally, Unix rats with or without treatment showed decreased weight gain and terminal body weight if compared to sham-operated rats. However, statistically significant trend was found only between sham-operated and Resveratrol-treated rats. The decrease in weight gain that has been demonstrated in Unix rats with or without treatment could not be attributed to the difference in food intake as we used the same standard chow with the same daily determined quantities (about 20 g/rat) for all rats in the three groups. However, animal studies showed that Unix decreased weight gain, which might occur due to lipodystrophy and inflammation seen in Unix rats. Moreover, the slight unsignificant decrease in weight gain in Resveratrol-treated rats compared to untreated Unix rats, which might be attributed to the ability of Resveratrol in decreasing weight gain that has been demonstrated before in nutritional studies [2].

Similarly, Unix rats treated with Resveratrol resulted in decreased oxidative and inflammatory changes, AChE expression and activity in hippocampal homogenates. We also reported that immune-stained brain sections from treated rats, showed increased SIRT1 expression. Besides its neuroprotective effect, Resveratrol preserved hippocampal and cognitive changes may be related to its nephroprotective and metabolic regulative function.

Unix rats showed spatial memory deficits manifested by significant decrease in spontaneous alternation in T maze test. Also, Unix rats showed changes in the species typical behaviour manifested by decreased burrowing activity in both the 2-h and 24-h burrowing tests. Species typical behaviour is in a close similarity to human daily activity, and therefore could be affected by the animal general condition and considered as sensitive evidence to track changes in chronic diseases. The decreased exploratory behaviour in Unix rats was shown by tendency to decrease rearing and square crossing in open field test. Increased anxiety was also shown by rats’ tendency to increase its latency to cross to white half dark–light box test, as well as their tendency to spend more time in the white half. Resveratrol administration ameliorated the memory deficit and anxiety, and restored the species typical and exploratory behaviours in Unix rats [15]. Anxiety behaviour was assessed in control rats as well to investigate the possible occurrence of stressful reaction because of the long period of oral gavage. The data, however, demonstrated the existence of anxiety behaviour significantly in Unix group.

The behavioural changes of Unix rats was associated with significant decrease in SIRT1 protein expression in immune-stained brain sections [25]. Decreased SIRT1 in Unix rats may be related to increased activity of renin angiotensin system (RAS), which inhibited AMPK (AMP protein kinase) activity. Inhibited AMPK leads to decreased AMP/ATP ratio, thus reducing the bioavailability of NAD^+^ which is important for SIRT1 activation [33]. Resveratrol is a well-known activator of both SIRT1 expression and activity. Moderate doses of Resveratrol increase levels of cAMP, increasing cellular calcium levels, thereby stimulating AMPK phosphorylation. As cellular NAD^+^ levels are found to be elevated under these conditions, this can be proposed as a mechanism by which Resveratrol activates SIRT1. The neuroprotective and behaviour corrective effects of Resveratrol may be linked to its ability to increase SIRT1 in brains of Unix rats [9].

Unix induced an inflammatory status in the hippocampus, manifested by increased mRNA expression of TNF-α, IL-1β, iNOS and IDO mRNA expression and decreased TGF-β and IGF 1 mRNA expression. The pro-inflammatory cytokines TNF-α and IL-1β are produced by M1 microglial cells via NFkβ signalling pathway and produce vicious inflammatory cycle, mediating neuro-degenerative processes. TGF-β and IGF 1 are produced by M2 microglial cells, promoting anti-inflammation, tissue healing and survival [26].

Both iNOS and IDO increased-mRNA expression is induced by the increased production of pro-inflammatory cytokines, TNF-α and IL-1β [8]. IDO activation breaks down tryptophan into neurotoxic tryptophan catabolites (mainly kynurenine), decreases serotonin synthesis and mediates cognitive and depressive disorders. iNOS also may be produced secondary to IDO activation and kynurenine production. iNOS produces excessive nitric oxide (NO), which reflects ongoing inflammatory and oxidative states, and contributes to apoptosis, and neurodegenerative processes [22].

The inflammatory status may be related to the significant decrease of SIRT1 protein and PPARγ mRNA expression in Unix rats. Decreased SIRT1 level in brains was associated with increased oxidative stress and microglial expression of inflammatory mediators, e.g. IL-1β and TNF-α, via NFkβ (nuclear factor kappa beta) upregulation [3]. Consecutively, decreased PPARγ expression is linked to increased inflammatory response and may be a consequence of SIRT1 deficiency. Downregulation of PPARγ is linked to brain energy dyshomeostasis, mitochondrial dysfunction and consequently neuro-inflammation [30].

Resveratrol administration significantly ameliorated the inflammatory state occurring in the hippocampus brains of Unix rats. Resveratrol mediates neuroprotection against inflammation by regulating M1/M2 microglia polarization via SIRT1-dependent deacetylation of PGC1α (peroxisome proliferator-activated receptor coactivator-1α) and activation of PPARγ [9], thus inhibiting NFkβ, ameliorating neuro-inflammation (TNF-α and IL-1β) and promoting anti-inflammatory expression (TGF-β and IGF 1) [5]. Consequently, Resveratrol-treated rats showed decreased mRNA expression of iNOS and IDO.

The dyslipidaemia occurring in Unix rats may be another cause for hippocampal changes. The brain lipogram is disturbed in dyslipidaemia. Oxidized lipids and increased MDA promoted endothelial cell apoptosis of the blood–brain barrier, thus altering its permeability to unsaturated fatty acids, increasing their content and promoting lipid synthesis in the brain [32]. The altered blood–brain barrier permits also the entry of urea toxins and systemic inflammatory cytokines to the brain [10]. Resveratrol ability to correct the metabolic dyshomeostasis is related to AMPK and SIRT1 activation, whereas its ability to decrease lipid peroxidation and MDA release is related to superoxide dismutase increased expression [9].

Though many studies showed decrease in AChE expression in chronic kidney disease [19], the present study showed that Unix was accompanied by a significant increase in both mRNA expression and activity assay of AChE in hippocampal homogenates. The increase in AChE activity may be linked to the dysmetabolism (decreased PPARγ and SIRT 1), increased lipid peroxidation (increased MDA) and oxidative stress occurred in Unix, rather than the renal dysfunction itself [16]. AChE increased activity may be related to the spatial memory defects in T maze test [4]. Resveratrol administration to Unix rats significantly ameliorated the upregulation of AChE mRNA expression. Schmatz et al. related the ability of Resveratrol to decrease AChE activity to its antioxidative properties [28].

CREB is a transcription factor that regulates the gene expression of neurotrophic factors, e.g. BDNF, by glial cells. BDNF is important for neuronal survival, synaptogenesis, neuroplasticity, long-term potentiation, memory consolidation and cognition. Disturbance in CREB transcriptional cascade results in oxidative stress, neuronal apoptosis and degeneration [21]. The present study showed that Unix tendency to reduce mRNA expression of CREB and BDNF in hippocampal homogenates was rescued by Resveratrol administration. CREB and BDNF reduction can be induced by inflammation, and Resveratrol-increased CREB and BDNF expression is linked to its anti-inflammatory property [15].

Based on the present findings and the safety profile of Resveratrol in daily recommended doses in humans [9], we suggested Resveratrol as a nephroprotective and neuroprotective agent that can be used in humans following nephron loss. Further studies addressing the cognitive changes following nephrectomy are warranted. The possible use of protective agents against renal and cognitive changes that may follow nephrectomy in humans should be thoroughly addressed.

## Summary

In conclusion, Unix rats not only showed renal and metabolic changes at 7 months post-nephrectomy, but also behavioural, memory and hippocampal molecular changes. Memory deficits, disturbed species typical behaviour, decreased explorative behaviour, and anxiety tendency were evident post-nephrectomy, together with increased oxidative, inflammatory changes and increased AChE activity in hippocampal homogenates. Immuno-stained brain sections of Unix rats showed decreased SIRT1 expression. However, Resveratrol administration to Unix rats ameliorated the renal, metabolic, behavioural and brain molecular changes.

## Abbreviations

Unix: Uninephrectomy
SIRT1: Silent information regulator 1
CKD: Chronic kidney disease
ESRD: End-stage renal disease
BDNF: Brain-derived neurotrophic factor
AChE: Acetyl cholinesterase
LDB: Light-dark box
TBW: Terminal body weight
MDA: Malondialdehyde
HDLc: HDL cholesterol
GAPDH: Glyceraldehyde 3 phosphate dehydrogenase
CREB: Cyclic AMP response element binding protein
IL-1β: Interleukin 1 beta
TNF-α: Tumour necrosis factor alpha
IDO: Indoleamine-2,3-dioxygenase
iNOS: Inducible nitric oxide synthase
IGF-1: Insulin growth factor 1
TGF-β: Transforming growth factor beta
PPAR γ: Peroxisome proliferator activated receptor gamma
